# Stressor-responsive central nesfatin-1 activates corticotropin-releasing hormone, noradrenaline and serotonin neurons and evokes hypothalamic-pituitary-adrenal axis

**DOI:** 10.18632/aging.100207

**Published:** 2010-10-12

**Authors:** Natsu Yoshida, Yuko Maejima, Udval Sedbazar, Akihiko Ando, Hideharu Kurita, Boldbaatar Damdindorj, Eisuke Takano, Darambazar Gantulga, Yusaku Iwasaki, Tomoyuki Kurashina, Tatsushi Onaka, Katsuya Dezaki, Masanori Nakata, Masatomo Mori, Toshihiko Yada

**Affiliations:** ^1^ Division of Integrative Physiology, Department of Physiology, Jichi Medical University School of Medicine, Shimotsuke, Tochigi 329-0498, Japan; ^2^ Division of Brain and Neurophysiology, Department of Physiology, Jichi Medical University School of Medicine, Shimotsuke, Tochigi 329-0498, Japan; ^3^ Department of Medicine and Molecular Science, Gunma University Graduate School of Medicine, Maebashi, Gunma 371-8511, Japan

**Keywords:** Nesfatin-1, Stress, c-Fos, Calcium, Paraventricular nucleus, CRH, HPA axis

## Abstract

A recently discovered satiety molecule, nesfatin-1, is localized in neurons of the hypothalamus and brain stem and colocalized with stress-related substances, corticotropin-releasing hormone (CRH), oxytocin, proopiomelanocortin, noradrenaline (NA) and 5-hydroxytryptamine (5-HT). Intracerebroventricular (icv) administration of nesfatin-1 produces fear-related behaviors and potentiates stressor-induced increases in plasma adrenocorticotropic hormone (ACTH) and corticosterone levels in rats. These findings suggest a link between nesfatin-1 and stress. In the present study, we aimed to further clarify the neuronal network by which nesfatin-1 could induce stress responses in rats. Restraint stress induced c-Fos expressions in nesfatin-1-immunoreactive neurons in the paraventricular nucleus (PVN) and supraoptic nucleus (SON) of the hypothalamus, and in the nucleus of solitary tract (NTS), locus coeruleus (LC) and dorsal raphe nucleus (DR) in the brain stem, without altering plasma nesfatin-1 levels. Icv nesfatin-1 induced c-Fos expressions in the PVN, SON, NTS, LC, DR and median raphe nucleus, including PVN-CRH, NTS-NA, LC-NA and DR-5-HT neurons. Nesfatin-1 increased cytosolic Ca^2+^ concentration in the CRH-immunoreactive neurons isolated from PVN. Icv nesfatin-1 increased plasma ACTH and corticosterone levels. These results indicate that the central nesfatin-1 system is stimulated by stress and activates CRH, NA and 5-HT neurons and hypothalamic-pituitary-adrenal axis, evoking both central and peripheral stress responses.

## INTRODUCTION

Nesfatin-1 is processed by cleavage of a precursor, NEFA/nucleobindin2 (NUCB2), and is distributed in the central nervous system (CNS) implicated in the regulation of feeding, including the hypothalamic paraventricular nucleus (PVN), arcuate nucleus (ARC), lateral hypothalamic area and supraoptic nucleus (SON) [1]. Double-labeling immunohistochemistry in these areas has revealed that nesfatin-1 is colocalized with feeding-related factors such as corticotropin-releasing hormone (CRH), oxytocin, proopiomelanocortin (POMC) and cocaine-amphetamine-regulated transcript (CART) [2-4]. Central administration of α-MSH increases NUCB2 mRNA in the hypothalamus. Anorectic effect of icv nesfatin-1 is mediated by the PVN oxytocin [5] to NTS POMC circuit [6]. Nesfatin-1 and oxytocin both suppresses food intake in Zucker-fatty rats with mutated leptin receptor, and leptin-induced satiety is unaltered by immunoneutralizing nesfatin-1 IgG [1,6]. These results suggest that nesfatin-1 induces anorexia in a leptin-independent and melanocortin-dependent manner [1,6]. It has also been reported that the anorectic action of nesfatin-1 involves the CRH pathway [7]. The CRH and melanocortin [10] pathways are implicated in stress.

Several studies have suggested a possible link of nesfatin-1 to stress responses. Nesfatin-1 is distributed in the stress-related areas, including the nucleus of solitary tract (NTS), locus coeruleus (LC) and raphe pallidus nucleus (RPa) [3] as well as PVN. Furthermore, nesfatin-1 is colocalized with CRH in the PVN and with noradrenaline (NA) and 5-hydroxytryptamine (5-HT) in the brain stem [3]. CRH in the PVN drives release of ACTH and corticosterone, evoking the hypothalamic-pituitary-adrenal (HPA) axis characteristically implicated in the stress responses, and NA and 5-HT modulate the HPA axis. Peripheral injection of 5-HT2C agonist increases NUCB2 mRNA in the hypothalamus [8]. Central administration of nesfatin-1 produces anorexi-genic and fear-related behaviors in several animal models [9]. Nesfatin-1 activates melanocortin pathway [1,6] implicated in stress responses [10]. Based on these observations, we hypothesized that nesfatin-1 could serve as a stress mediator. Nesfatin-1 is also localized in the peripheral tissues including stomach [11,12], pancreas [13,14] and adipose tissue [15], though its function remains largely unknown.

This study aimed to clarify whether the central nesfatin-1 system and/or the plasma nesfatin-1 level is activated by restraint stress, to specify the central areas and neuron species targeted by stress and icv nesfatin-1, and to examine whether icv nesfatin-1 stimulates HPA axis in rats. c-Fos protein expression was examined to detect neuronal activation. Direct effects of nesfatin-1 on specified neurons were determined by measurement of cytosolic Ca^2+^ concentration ([Ca^2+^]_i_) in isolated neurons combined with immuno-cytochemistry. We here show that the central nesfatin-1 is stimulated by stressor and that icv nesfatin-1 activates CRH, NA and 5-HT neurons and HPA axis.

## RESULTS

### Nesfatin-1 was colocalized with TH in the NTS and LC

**Figure 1.**
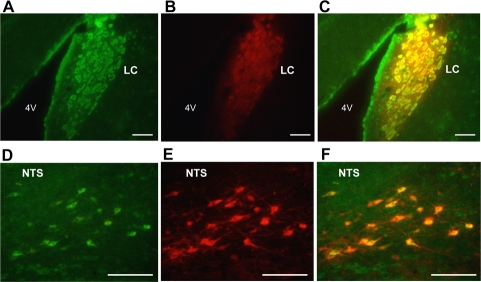
Dual immunofluorescence histochemistry for nesfatin-1 and tyrosine hydroxylase (TH). (**A-C**) Neurons immunoreactive (IR) to nesfatin-1 (green) (**A**) and TH (red) (**B**), and their merged image (C) in the LC. (**D-F**) Neurons IR to nesfatin-1 (green) (**D**) and TH (red) (**E**), and their merged image (**F**) in the NTS. Colocalization of nesfatin-1 with TH is seen by yellow color in (**C**) and (**F**). Scale bar, 100 μm. 4V, forth ventricle.

Double fluorescence immunohistochemistry in the LC showed that immunoreactivity for TH (Figure 1A) and that for nesfatin-1 (Figure 1B) overlapped extensively in neurons in the LC (Figure 1C), demonstrating colocalization of two molecules in the LC neurons. Similarly, immunoreactivities for TH (Figure 1D) and nesfatin-1 (Figure 1E) overlapped in neurons in the NTS (Figure 1F), confirming previous reports [3,17].

### Restraint stress induced c-Fos expressions in nesfatin-1 neurons in the hypothalamus and brain stem but failed to alter plasma nesfatin-1 concentrations

**Figure 2.**
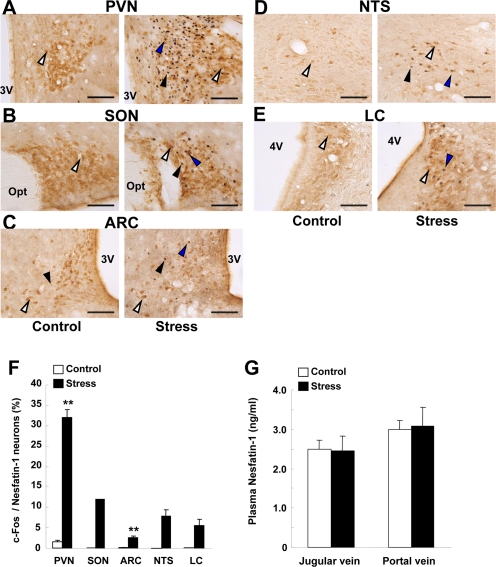
c-Fos expressions on nesfatin-1 neurons in several brain areas and plasma nesfatin-1 levels after restraint stress. (**A-E**) Double-immunohistochemical staining of c-Fos (black) and nesfatin-1 (brown) in the PVN (A), SON (**B**), ARC (**C**), NTS (**D**) and LC (**E**) in control conditions (left panels) and after restraint stress (right panels). White arrows indicate nefatin-1-IR neurons, black arrows c-Fos-IR neurons, and blue arrows both-IR neurons. (**F**) Incidence of c-Fos-IR neurons in nesfatin-1-IR neurons, as expressed by percentage. Number of c-Fos was significantly greater in stress than control conditions in the PVN and ARC, and found only in stress conditions in SON, NTS and LC. **p < 0.01 vs. control. (**G**) Nesfatin-1 concentrations in the plasma of jugular vein and portal vein were not different between control and stress conditions.

Exposure of rats to restraint stress for 60 min, as compared to control groups without stress, induced c-Fos expression in the areas of the hypothalamus and brain stem where nesfatin-1 is localized. In the PVN, the number of c-Fos immunoreactive (IR) neurons in stress groups (180.3 ± 13.5 neurons/section, n = 12 slices) were significantly (P < 0.01) greater than those in control groups (5.8 ± 1.0 neurons/section, n = 13) (Figures 2A and G). In these c-Fos-expressing neurons, 47.1 ± 3.2% was IR to nesfatin-1. Conversely, 32.0 ± 2.0% of nesfatin-1-IR neurons expressed c-Fos in stress groups, while only 1.5 ± 0.4% in control groups (Figures 2A and F). Stress also induced significant c-Fos expression in nesfatin-1 neurons in the SON, ARC, NTS and LC (Figures 2B-E and F). c-Fos expression was significantly increased (9.3 ± 2.0, n = 17, in stress group vs. 0.2 ± 0.1, n = 22, in control group, P < 0.01) in the DR. The nesfatin-1 level in the blood from jugular vein in rats after exposure to restraint stress for 15 min (2.5 ± 0.4 ng/ml, n = 3) was not different from that in control rats (2.5 ± 0.2 ng/ml, n = 3), and the nesfatin-1 level in the blood from portal vein was not different between stress (3.1 ± 0.5 ng/ml, n = 3) and control groups (3.0 ± 0.2 ng/ml, n = 3) (Figure 2G).

### Icv administration of nesfatin-1 induced c-Fos expression in stress-related nuclei

**Figure 3.**
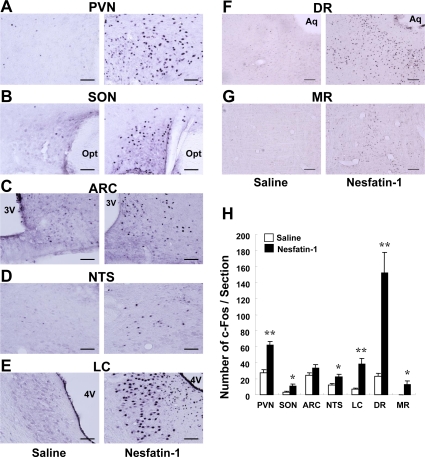
c-Fos expressions in several brain areas after icv administration of nesfatin-1. The number of c-Fos was significantly greater in the group treated with icv nesfatin-1 (0.5 nmol / 5 μl) than in control saline-treated group in the PVN (**A,H**), SON (**B,H**), NTS (**D,H**), LC (**E,H**), DR (**F,H**) and MR (**G,H**), while no difference was observed between two groups in the ARC (C,H). *p < 0.05, **p < 0.01 vs. control.

We next examined which of the brain areas could be activated by icv nesfatin-1. Injection of nesfatin-1 into 3v (0.5 nmol/5 μl) significantly increased the number of neurons that expressed c-Fos in the PVN (nesf-1; 62.3 ± 4.2, n = 22 slices, vs. saline; 27.2 ± 3.8, n = 20 slices; Figure 3-A,H), SON (nesf-1; 10.7 ± 3.0, n = 31, vs. saline; 3.2 ± 1.0, n = 27; Figure 3-B,H), NTS (nesf-1; 22.1 ± 3.5, n = 23, vs. saline; 12.2 ± 1.7, n = 20; Figure 3-D,H), LC (nesf-1; 38.0 ± 7.5, n = 32, vs. saline; 7.1 ± 1.6, n = 15; Figure 3-E,H), dorsal raphe nucleus (DR) (nesf-1; 152.2 ± 25.2, n = 29, vs. saline; 23.0 ± 3.7, n = 25; Figure 3-F,H) and median raphe nucleus (MR) (nesf-1; 12.5 ± 4.9, n = 18, vs. saline; 0.1 ± 0.1, n = 14; Figure 3-G,H). Nesfatin-1 failed to induce c-Fos in the ARC (Figure 3-C,H).

### Icv nesfatin-1 induced c-Fos expression in neurons containing stress-related neurohormones

**Figure 4.**
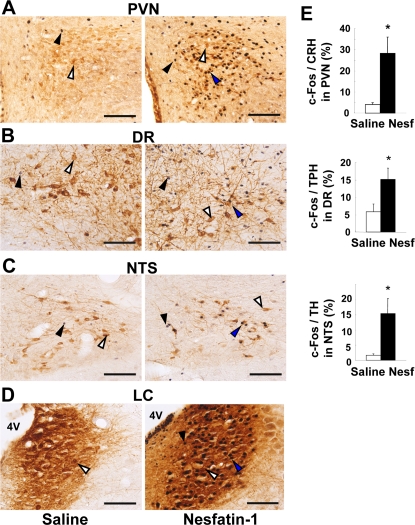
c-Fos expressions in stress-related neurons after icv administration of nesfatin-1. (**A-D**) Double-immunohistochemical staining of c-Fos (black) together with CRH (brown) in PVN (**A**), with tryptophan hydroxylase (TPH) in DR (**B**), with TH in NTS (**C**) and LC (**D**) after icv saline (left panels) and nesfatin-1 (right panels). (**E**) Incidence of c-Fos-IR neurons in CRH-IR, TPH-IR and TH-IR neurons in the corresponding areas, respectively. Scale bars, 100 μm. 4V, forth ventricle. White arrows indicate CRH-, TPH- or TH-IR neurons, black arrows c-Fos-IR neurons, and blue arrows both-IR neurons. *p < 0.05 vs. saline.

We intended to identify the target neurons for central nesfatin-1 by double immunohistochemistry. Icv administration of nesfatin-1 induced c-Fos expression in CRH neurons in the PVN (Figure 4A): the incidence of c-Fos expression in CRH neurons was significantly (P < 0.05) higher after nesfatin-1 treatment (28.3 ± 7.7%, n = 12 slices) compared to saline treatment (3.9 ± 1.0%, n = 9) (Figure 4E). Vice versa, a large fraction of the c-Fos-expressing neurons after icv nesfatin-1 (44.2 ± 6.7%, n = 12) contained CRH. These data indicated that CRH neuron was a major target of icv nesfatin-1 in the PVN. In the DR, icv nesfatin-1 increased the incidence of c-Fos expression in tryptophan hydroxylase (TPH)-IR neurons (nesf-1; 15.1 ± 3.3%, n = 29 vs. saline; 5.8 ± 2.2%, n = 27, P < 0.05) (Figures 4B and E). Vice versa, 15.3 ± 2.3% of the neurons that expressed c-Fos after icv nesfatin-1 contained TPH (n = 24). These data indicated that 5-HT neuron was a target of icv nesfatin-1 in the DR. In the NTS, icv nesfatin-1 increased the incidence of c-Fos expression in TH-IR neurons (nesf-1; 15.4 ± 4.9%, n = 24, vs. saline; 1.6 ± 0.5%, n = 21, P < 0.05) (Figures 4D and E). Vice versa, 23.9 ± 4.2% (n = 22) of the neurons that expressed c-Fos after icv nesfatin-1 contained TH. These data indicated that noradrenalin neuron was a substantial target of icv nesfatin-1 in the NTS. In the LC, nesfatin-1 induced a marked c-Fos expression in TH-IR neurons (Figure 4E). However, it was difficult to count c-Fos-expressing TH-IR neurons since neurons extensively overlapped to each other. In addition, icv nesfatin-1 also increased the incidence of c-Fos expression in oxytocin neurons in the PVN (data not shown), confirming our previous report [6].

### Nesfatin-1 increased [Ca^2+^]_i_ in single CRH neurons in the PVN

**Figure 5.**
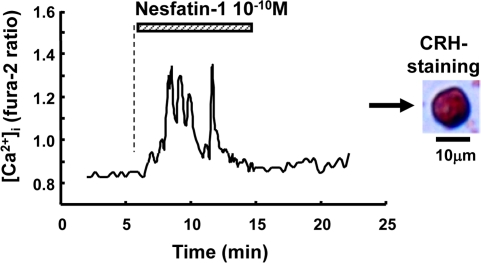
The effect of nesfatin-1 on [Ca^2+^]_i_ in CRH neurons in the PVN. Administration of 10^-10^ M nesfatin-1 increased [Ca^2+^]_i_ in a single neuron isolated from PVN (left panel) that was subsequently shown to be IR to CRH (light panel). The bar above the tracing indicates the period of nesfatin-1 administration. Four out of 16 (25%) neurons that responded to nesfatin-1 were CRH-IR neurons.

The results that icv nesfatin-1 induced c-Fos in CRH neurons raised a question whether nesfatin-1 could directly interact with this neuron. Administration of 10^-10^ M nesfatin-1 increased [Ca^2+^]_i_ in a single neuron isolated from the PVN (Figure 5A, left), and the neuron was subsequently shown to be immunoreactive to CRH (Figure 5A, right). Nesfatin-1 at 10^-10^ M increased [Ca^2+^]_i_ in 4 out of 16 PVN CRH-IR neurons (25%). The results revealed that nesfatin-1 directly interacts with and increases [Ca^2+^]_i_ in the PVN CRH neurons.

**Figure 6.**
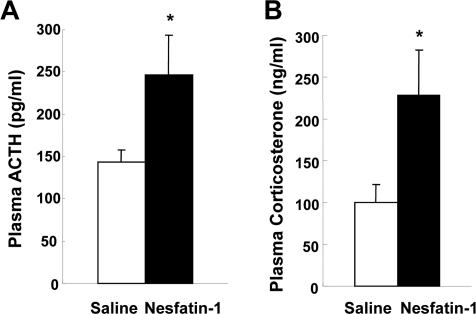
The effects of nesfatin-1 on plasma ACTH and corticosterone concentrations. Plasma ACTH levels at 10 min (**A**) and corticosterone levels at 15 min (**B**) after icv administration of nesfatin-1 (0.5 nmol/5 μl) were increased compared with saline administration. *p < 0.05 vs. saline.

### The effect of central nesfatin-1 on plasma ACTH and corticosterone levels

The result that nesfatin-1 activated CRH neurons suggested a possibility that this action is linked to the HPA axis. Icv nesfatin-1 significantly increased the plasma ACTH levels at 10 min after administration, compared to icv saline (247.1 ± 46.8 pg/ml (n = 15) with nesfatin-1 vs. 143.4 ± 14.7 pg/ml (n = 15) with saline, p < 0.05). Icv nesfatin-1 also significantly increased the plasma corticosterone levels at 15 min after administration, compared with control (228.0 ± 54.9 ng/ml (n = 11) with nesfatin-1 vs. 100.4 ± 20.6 ng/ml (n = 11) with saline, p < 0.05). The elevated levels of ACTH and corticosterone returned to the basal levels at 30 and 60 min after administration, respectively (data not shown). These data demonstrate that icv nesfatin-1 evokes CRH-ACTH-corticosterone pathway HPA axis.

## DISCUSSION

The present study has demonstrated that the central nesfatin-1 system but not the plasma nesfatin-1 level is activated by restraint stress and that icv nesfatin-1 activates CRH, noradrenaline and 5-HT neurons and increases plasma ACTH and glucocorticoid levels.

In this study, restraint stress induced abundant c-Fos expressions in nesfatin-1 neurons in the stress-related brain areas, PVN, SON, NTS and LC, confirming the recent report by Goebel et al. [17]. More importantly, we have found for the first time that centrally administered nesfatin-1 induced significant c-Fos expression in PVN, SON, NTS, LC, DR and MR, including more specifically the CRH neurons of PVN, the NA neurons of NTS and LC, and the 5-HT neurons of DR. In addition, we have first demonstrated that nesfatin-1 directly interacted with and increased [Ca^2+^]_i_ in the CRH neurons isolated from PVN, and that centrally administered nesfatin-1 by itself, with no added stress, increased plasma ACTH and corticosterone levels. These results collectively demonstrate that the nesfatin-1 neuronal system in the hypothalamus and brain stem respond to stressor and evokes central stress responses leading to activation of the HPA axis. This mechanism may underlie the nesfatin-1-induced stress-related behaviors [18].

In the present study, we confirmed previous report that nesfatin-1 is colocalized with NA in the NTS [3] and added that nesfatin-1 is also colocalized with NA in the LC, the area of the largest source of NA neurons. We also confirmed the finding by Goebel et al. [17] that the nesfatin-1 neurons in the PVN, SON, LC, and NTS are activated by stress and added that those in the ARC are also activated by stress and that plasma nesfatin-1 concentrations are altered neither by restraint stress nor by a stronger stress of water immersion-restraint (data not shown). These results suggest that the central but not peripheral nesfatin-1 is involved in stress responses.

We showed that icv injection of nesfatin-1 induces c-Fos expression in CRH, NA and 5-HT neurons in the PVN, NTS and DR, respectively. In particular, a large fraction (28.3%) of CRH neurons expresses c-Fos in the PVN after icv nesfatin-1. Furthermore, nesfatin-1 directly interacts with and increases [Ca^2+^]_i_ in a substantial fraction (25%) of CRH neurons isolated from the PVN, in accord with previous report that nesfatin-1 changes electrical activities of CRH neurons [19]. Thus, nesfatin-1 activates CRH neurons both in vivo and ex vivo. These results demonstrate that nesfatin-1 targets the PVN CRH neurons.

In the present study we have demonstrated that icv nesfatin-1 increases plasma ACTH and corticosterone concentrations confirming the previous report [20]. After icv administration of nesfatin-1, plasma ACTH and corticosterone levels increased at 10 and 15 min and returned toward basal levels at 30 and 60 min, respectively. This time course of plasma ACTH levels fits with the reports that plasma ACTH concentrations increase maximally at 5-15 min after administration of several stress-related neuropeptides [21-23] and under restraint stress conditions [24]. On the other hand, our data of the time course of plasma corticosterone levels appear somewhat shorter-lasting than those previously reported in which the plasma corticosterone reaches and keeps maximum levels during 30-120 min, and then declines to the basal levels [23,25]. Our data of relatively shorter-lasting increase of plasma corticosterone by icv nesfatin-1 may suggest that the central nesfatin-1 is implicated in the initiation or the early phase of stress responses.

It has been shown that nesfatin-1 is localized not only in the central nervous system but in the peripheral tissues including stomach [11,12], pancreas [13,14], and adipose tissue [15]. The present study indicated that stress induced c-Fos in the nesfatin-1 neurons without altering plasma nesfatin-1 levels. The results suggest that stress involves the central but not circulating nesfatin-1. The precise neural mechanism by which nesfatin-1 neurons mediate stress responses remain to be elucidated. However, the nesfatin-1 neurons in the stress-activated nuclei such as NTS, LC and SON may project to the PVN CRH neurons. Alternatively, stress-activated nesfatin-1 neurons in the PVN may activate CRH neurons via a paracrine and/or ultrashort feedback regulation, which is supported by that nesfatin-1 is localized in secretory vehicles in PVN neurons [6].

We found that central nesfatin-1 also activates NA neurons in the NTS and LC and 5-HT neurons in the DR of the brain stem. It has been documented that these NA and 5-HT neurons project to PVN [26-28] including CRH neurons [29-31], being relayed to activation of HPA axis [32-35]. Our data together with these documents suggest that the nesfatin-1-activated NA and 5-HT neurons project to and stimulate PVN CRH neurons and that this pathway partly contributes to the nesfatin-1 activation of HPA axis.

We found that icv nesfatin-1 induces c-Fos expression in 5-HT neurons. Vice versa, peripheral injection of a 5-HT receptor agonist reportedly increases NUCB2 mRNA expressions in the hypothalamus [8]. These findings suggest a bidirectional interaction between 5-HT and nesfatin-1 neurons.

In summary, this study presents evidence to support that stressor-responsive nesfatin-1 neurons in the hypothalamus and brain stem activate the CRH, NA and 5-HT neurons and evoke HPA axis, thus inducing stress responses. Central nesfatin-1 system could provide a potential therapeutic target for treating stress-related disorders in humans.

## METHODS

### Animals

Male Sprague Dawley rats aged 7-8 weeks were housed in individual cages and maintained under a controlled environment (12h/12h dark/light cycle, lights on at 07:30) and allowed free access to conventional food (CE-2; Clea, Osaka, Japan) and water ad libitum. All procedures were performed in accordance with institutional guidelines for Animal Care at Jichi Medical University.

### Restraint stress

Rats were wrapped in a wire mesh and kept in their cages for 15 or 60 min. Control rats were left in their cages without any treatment. At the termination of the stress period, animals were released from a wire mesh. The stress was given in day time (10:00-14:00h).

### Intracerebroventricular cannulation

Rats were anesthetized by injection of Avertin (Tribromoethanol, 200 mg/kg, ip) and placed in a stereotaxic frame (DAVIO Kopf Instruments, Tujunga, CA), and a stainless steel guide cannula (26 gauge) was inserted into the brain with the tip in the third ventricle (3v); 2.5 mm posterior from the bregma and 8mm below the skull). Rats were allowed 9-11 days to recover from the surgical procedure and were handled daily to minimize non specific stress responses. Substances were administered into 3v via a stainless steel injector (30 gauge) through the guide cannula. Rats received an administration of 0.5 nmol nesfatin-1 in 5 μl of 0.9 % NaCl into 3v. Experiments were carried out during the light phase (for immuno-histochemical study at 10:00-14:00h, and for blood collection at 10:00-11:00h). Hypothalamic sections were histologically examined at the end of the study, and the placement of the cannula was verified. The rats in which cannulae were outside 3v were excluded from the data analysis.

### Tissue preparation for immunohistochemistry

At 2 h after 60 min restraint stress and at 2 h after icv administration of nesfatin-1, rats were deeply anesthetized with urethane (1 g/kg, ip) and perfused with saline containing heparin (20 U/ml) and then 4% paraformaldehyde in 0.1 M phosphate buffer. The brains were immediately removed, postfixed in the same fixative overnight at 4°C, and then immersed in PBS containing 30% sucrose for at least 2 days at 4°C. The brains were frozen on dry ice and kept at -80°C until sectioning. Frozen frontal sections (40μm) were prepared with a freezing microtome and collected at 240 μm intervals. In immunohistochemical studies, each quantitative analysis was performed in 9 to 32 sections collected from 3 animals.

### Double-labeling immunohistochemistry for nesfatin-1 and TH

Fixed brain sections were prepared during 10:00-14:00h as described above. Secitons were rinsed in PBS, and then incubated in 0.3% TritonX containing PBS (PBST) for 30 min and then blocked for 30 min in 2% BSA-containing PBST. Finally, sections were incubated with rabbi anti-nesfatin-1 antibody (1:1000) (Oh-I et al. 2006) and mouse monoclonal anti-tyrosine hydroxylase clone TH2 antibody (Sigma Aldrich, MO ;1:4000) overnight at 4°C. Then the sections were rinsed and incubated with secondary antibodies, Alexa 488 goat anti-rabbit IgG (Invitrogen Co., CA ; 1:500) and Alexa 594 goat anti-mouse IgG (Invitrogen Co., CA ; 1:500) for 40 min. Slices were then rinsed, mounted on slides, and coverslipped with fluorescent mounting medium (DakoCytomatin, Carpinteria, CA). Fluorescence images were acquired with a BX50 microscope and a DP50 digital camera (Olympus, Tokyo, Japan). Using Photoshop (Adobe, San Jose, CA), brightness and contrast were adjusted, and fluorescence photographs were combined to visualize double-labeled cells by the screen blending mode.

### c-Fos immunohistochemistry

Fixed brain tissues were prepared as described above. Sections were rinsed in PBS, and then incubated in PBS containing 0.3% H_2_O_2_ for 30 min to quench endogenous peroxidase. After the rinse, sections were incubated in PBST for 30 min and then blocked for 30 min in 2% BSA-containing PBST. Finally, sections were incubated with rabbit anti c-Fos antibody (Ab-5; Calbiochem, CA; 1:40,000) overnight at 4°C. Then the sections were rinsed and incubated with biotinylated goat anti-rabbit IgG (Vector Laboratories, CA; 1:500) for 30 min and then with ABC reagent (Vector Laboratories, CA; ABC KIT) for 30 min. After the rinse in PBS and 0.05 M Tris-HCl buffer (pH7.4), color was developed with a nickel-diaminobenzidine (DAB) solution (0.2 g/liter DAB and 0.015 % H_2_O_2_ in 0.05 M Tris-HCl buffer (pH7.4)) for 5 min. Slices were mounted on slides and coverslipped with Entellan new (Merck, Darmstadt, Germany).

### Double-labeling immunohistochemistry for c-Fos and nesfatin-1, CRH, NA or 5-HT

Double-labeling immuno-histochemistory for c-Fos together with nesfatin-1, CRH, NA or 5-HT was performed by the procedures stated above and according to Kohno et al. [2]. Rabbit anti nesfatin-1 antibody (1:5000) [1], rabbit anti CRH antibody (BACHEM, CA. 1:5000), mouse monoclonal anti-TH2 antibody (Sigma Aldrich, 1:4000) or mouse monoclonal anti-tryptophan hydroxylase (Ab-1) antibody (Calbiochem, 1:800) was used for each neuropeptide staining.

### Measurement of [Ca^2+^]_i_ in single PVN neurons

[Ca^2+^]_i_ was measured by radiometric fura-2 microfluorometry in combination with digital imaging as previously reported [6]. Briefly, following incubation with 2 μmol/l fura2/AM (Dojin Chemical, Kumamoto, Japan) for 1 h at 18C, the cells were mounted in a chamber and superfused with HKRB at 1 ml/min at 33°C. Fluorescence images due to excitation at 340 and 380 nm were detected every 8.0 s with an intensified charge-coupled device (ICCD) camera, and the ratio (F340/F380) image was produced by an Argus-50 system (Hamamatsu Photonics, Hamamatsu, Japan). When [Ca^2+^]_i_ increased within 5 min after addition of nesfatin-1 with the ratio changes greater than 0.3, they were considered responses. Data were taken from the cells that were identified as neurons by the criteria reported previously [16].

### Effect of stress on plasma nesfatin-1 levels

After restraint stress for 15 min given in day-time (10:00-14:00h), rats were anaesthetized by injection of urethane (1 g/kg, ip) and then blood were collected from jugular vein or portal vein. After addition of 1/10 amounts of 1 N HCl to the plasma, plasma was stored at -80°C until the measurement of nesfatin-1. The plasma nesfatin-1 concentrations were measured with a RIA kit (Phoenix Parmaceuticals, Inc., CA).

### Effect of 3v nesfatin-1 administration on plasma ACTH and corticosterone levels

At 10 min (for ACTH) or 15 min (for corticosterone) after icv administration of nesfatin-1, rats were decapitated and blood was collected into tubes containing EDTA and aprotinin. Plasma was separated from blood and stored at -80°C until the measurement of ACTH or corticosterone. The plasma ACTH concentrations were measured with a RIA kit (Mitsubishi Chemical Medience Co., Tokyo, Japan) and plasma corticosterone concentrations with an ELISA kit (Assaypro, MO). These studies were performed during day-time (10:00-11:00h).

### Statistics

All data were presented as the means ± SEM (n = number of animals or slices). Statistical analysis was carried out using Student's t test for two-group experiments. The statistical significance level was set at P < 0.05.
